# Twitter data from the 2019–20 Australian bushfires reveals participatory and temporal variations in social media use for disaster recovery

**DOI:** 10.1038/s41598-022-21265-6

**Published:** 2022-10-08

**Authors:** R. Ogie, A. Moore, R. Wickramasuriya, M. Amirghasemi, S. James, T. Dilworth

**Affiliations:** 1 SMART Infrastructure Facility, Faculty of Engineering and Information Sciences, University of Wollongong, Northfields Ave, Wollongong, NSW 2522 Australia; 2 School of Humanities and Social Inquiry, Faculty of the Arts, Social Sciences and Humanities, University of Wollongong, Northfields Ave, Wollongong, NSW 2522 Australia; 3 School of Public Health and Preventive Medicine, Faculty of Medicine, Nursing and Health Sciences, Monash University, Notting Hill, VIC 3168 Australia; 4 School of Geography and Sustainable Communities, Faculty of the Arts, Social Sciences and Humanities, University of Wollongong, Northfields Ave, Wollongong, NSW 2522 Australia

**Keywords:** Natural hazards, Sustainability, Psychology and behaviour

## Abstract

Social media platforms have proved to be vital sources of information to support disaster response and recovery. A key issue, though, is that social media conversation about disasters tends to tail off after the immediate disaster response phase, potentially limiting the extent to which social media can be relied on to support recovery. This situation motivates the present study of social media usage patterns, including who contributes to social media around disaster recovery, which recovery activities they contribute to, and how well that participation is sustained over time. Utilising Twitter data from the 2019–20 Australian bushfires, we statistically examined the participation of different groups (citizens, emergency agencies, politicians and others) across categories of disaster recovery activity such as donations & financial support or mental health & emotional support, and observed variations over time. The results showed that user groups differed in how much they contributed on Twitter around different recovery activities, and their levels of participation varied with time. Recovery-related topics also varied significantly with time. These findings are valuable because they increase our understanding of which aspects of disaster recovery currently benefit most from social media and which are relatively neglected, indicating where to focus resources and recovery effort.

## Introduction

Social media is a potentially invaluable technology for supporting disaster management activities, including disaster preparedness, response, and recovery^1^. Disasters such as floods and bushfires pose a huge threat to lives and property across the world. With climate change, the frequency and intensity of these disasters are increasing, resulting in enormous social and economic costs that hamper efforts in making human settlements resilient and sustainable^2^. There is a vital role for information and communication technology such as social media to address disaster risks and build resilient communities^3,4^. The need for these social networking technologies has been made clearer by lockdowns and social distancing accompanying the COVID-19 global pandemic. According to Ogie et al.^5^, “the phenomenal growth in the richness and diversity of time-critical information shared on social media platforms during natural disasters provides a unique opportunity to harvest large-scale spatio-temporal data of immense value to emergency managers”.

However, social media use in disaster recovery is still an under-researched field. There are only a few studies that have explored social media use for disaster recovery, with some notable studies focusing on bushfires^6,7^, floods^8,9^, earthquakes^10–12^, and hurricanes^13,14^. More research is still required to help design strategies to consistently harness social media data for improved disaster recovery outcomes. Compared with other phases of disaster management, recovery is the least studied and rests on weak theoretical foundations, requiring further research to explore how social media can better serve to improve disaster outcomes^8,14^. This research gap recently inspired a comprehensive literature review, which found that social media could contribute to several aspects of disaster recovery, including (1) donations and financial support, (2) solidarity and social cohesion, (3) post-disaster reconstruction and infrastructure services, (4) socioeconomic and physical wellbeing, (5) information support, (6) mental health and emotional support, and (7) business & economic activities^15^.

One key issue, though, is that unlike disaster response that occurs during the active phase of natural hazards when public interest and social media engagement is the highest, disaster recovery lingers well beyond the active phase to a time when interest in the event gradually drops. Previous studies have reported that the reduction in the volume of messages could potentially limit social media’s role in disaster recovery^8,16^. This situation motivates the present investigation of social media patterns, including who contributes to the generation of social media data for disaster recovery, what aspects of disaster recovery they contribute to, and how well that participation is sustained over time.

Our focus on user categories draws on social practice theory, especially the concepts of *field* and *habitus* as developed in particular by Bourdieu^17,18^, and *social role* theory, as in the work of Parsons^19^. These approaches help explain why people within the same group (e.g., social class, profession, industry, culture, etc.) tend to exhibit similar behaviour and share a common worldview, due to culturally and socially acquired norms of acting and thinking. The concept of habitus describes the lasting dispositions that individuals or groups develop and reproduce generationally and explains why actions are not simply intentional individual behaviours^20^. Linking this to our research, *habitus* shows how groups determine what is reasonable or unreasonable social participation within their *field* of practice^20,21^. On this basis, we posit that different social media user groups are likely to exhibit distinct social media participation patterns across different recovery activities.

To explore these patterns, we conducted a case study using Twitter data from the 2019–20 Australian ‘Black Summer’ Bushfires. The bushfires started in August 2019, peaked in the December–January period and, by the end of the fires in March 2020, had destroyed an estimated 12.6 million hectares, damaged over 3000 homes and 7000 other structures^22^. These fires have been described as Australia's costliest natural disaster, with property and economic losses estimated to be over 103 billion Australian dollars^23^. The fires directly caused the deaths of 33 people and over one billion native animals^22^. A further 417 human deaths occurred due to smoke inhalation, with 80% of the Australian population estimated to have been directly or indirectly affected by the fires^22,24^. The bushfires attracted substantial social media engagement^25,26^, prompting this case study of social media use in disaster recovery. As part of a broader project involving interviews with community participants regarding their use of social media, the present paper reports on the participation of a wide range of user groups during the bushfires and subsequent recovery period. Below are the research questions and hypotheses.

Research questions

Q1: How do the bushfire recovery topics on Twitter vary with time?
Q2: How do the categories of users that post bushfire recovery messages on Twitter vary with time?
Q3: How do the bushfire recovery topics on Twitter vary with change in the categories of users that post the messages?

We hypotheses as follow:

### H1

There is a relationship between the categories of bushfire recovery topics posted on Twitter and the timing of the messages.

### H2

There is a relationship between the categories of users that are posting bushfire recovery messages on Twitter and the timing of the messages.

### H3

There is a relationship between the categories of users that are posting bushfire recovery messages on Twitter and the recovery topics that are represented in the posts.

## Methods

**Figure 1:**
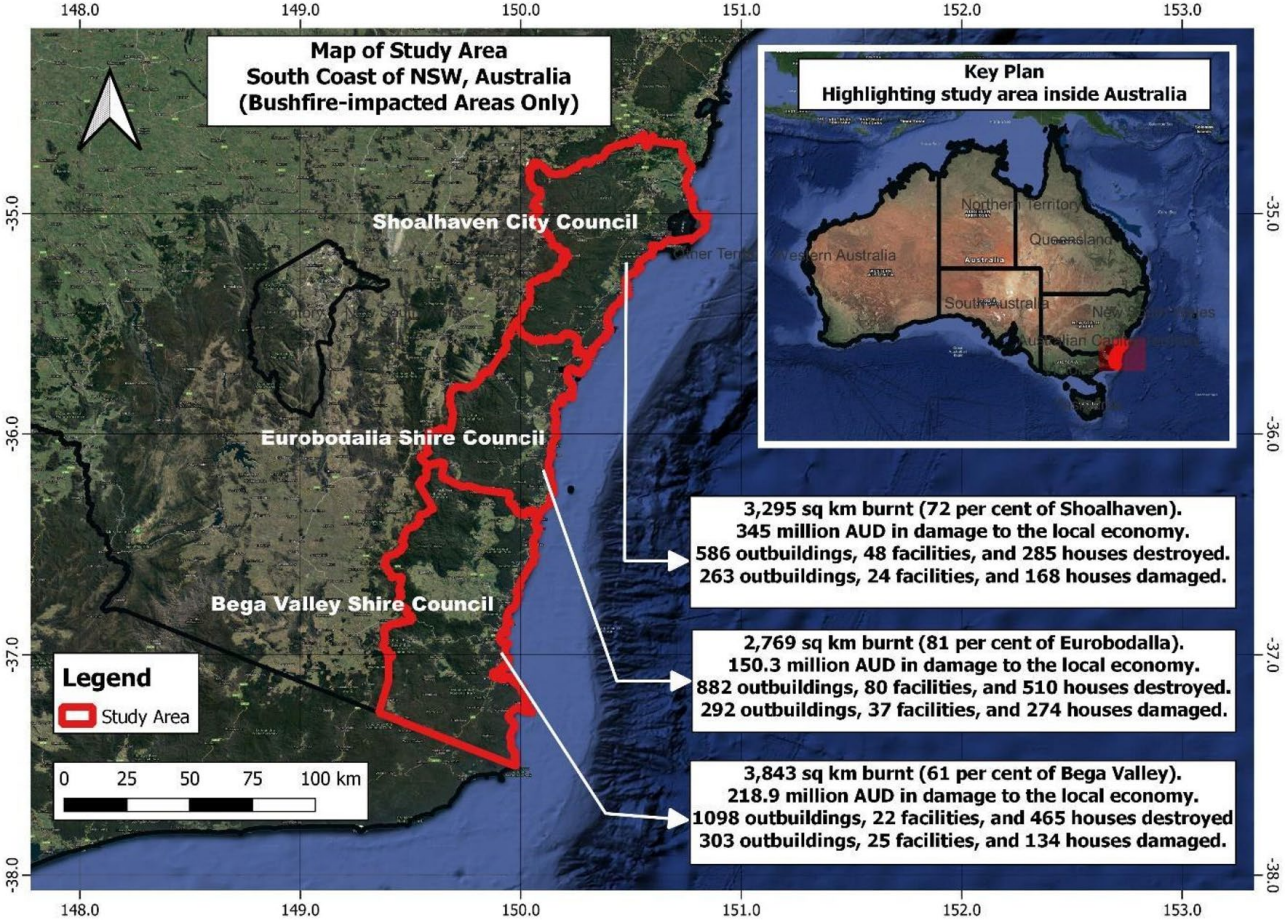
Study area: bushfire-impacted local government areas in the south coast region of New South Wales (NSW), Australia. (Source of Statistics: National Bushfire Recovery Agency^29^). The map was created by the first author using the free and opensource Quantum geographic information system (QGIS v.3.22.3 'Białowieża'; QGIS Development Team, https://www.qgis.org/en/site/). The satellite imagery is Google Hybrid Maps layer accessible via the QGIS v.3.22.3 software. The digital boundary files for Australia was sourced from the Australian Bureau of Statistics (https://www.abs.gov.au/statistics/standards/australian-statistical-geography-standard-asgs-edition-3/jul2021-jun2026/access-and-downloads/digital-boundary-files).

To study social media use in disaster recovery, the Full-Archive Search API was used in December 2020 to retrieve tweets about the 2019–20 Australian bushfires. As a study of the entire recovery phase would take many years and exceed our resources, our focus was on early recovery. Following influential work showing that the disaster cycle and its phases are best considered open-ended and cyclical^27,28^, we did not want to define a specific date on which we thought recovery might begin; rather, we wanted to see whether a phase shift into recovery could be evident from tweet data showing changes in the topics and groups that appeared more frequently in relevant tweets over time. Therefore, tweets were collected for the period of October 1, 2019 (active emergency phase) to August 31, 2020 (5 to 6 months after there were active bushfires in the area of concern). The study focused on a specific geographical region, namely the South Coast of New South Wales (NSW), Australia, from the Shoalhaven to the Victorian border (see Fig. 1). A total of 200,017 tweets were initially retrieved using a keyword search strategy. Since only about one per cent of tweets are geotagged, the search strategy was aimed at retrieving tweets containing the word, ‘fire’ in combination with one or more bushfire-impacted NSW South Coast locations, namely Cobargo, Shoalhaven, Bega, Eurobodalla, Mogo, Malua Bay, Kangaroo Valley, Batemans Bay, and Eden. The hashtags and keywords of the same entity were included in the search strategy. Tweets that used the word, ‘fire’ in combination with NSW or Southcoast (e.g., #NSWfires) were also included if they generally conveyed useful information about the NSW bushfires and were not intended solely for any other specific locations outside the study area.

The 200,017 retrieved tweets were assigned to members of the research team to identify recovery-specific content, eliminating posts that were trivial or not related to bushfire recovery. Tweets were considered to be relevant if they related to one or more aspects of disaster recovery, as adopted from previous studies and described in Table 1^15^. This resulted in the identification and extraction of 61,645 tweets related to bushfire recovery. Although bot activities have been found to be prevalent in online public conversions, we did not detect any bot accounts after a careful examination of a few suspicious accounts that were initially identified by botometer within our sample data (for more details about botometer and bot detection, see^30^ and^31^. However, we found 283 tweets posted from suspended/closed accounts that have no user information. We suspect that some of these accounts may well be bots that were detected by Twitter and consequently shut down. Hence, the 283 tweets were removed, leaving a total of 61,362 tweets for analysis. Based on descriptions associated with user accounts, usernames, and/or researcher familiarity with the user’s public identity, each tweet was categorised into one of several user categories (described in Table 2). Our user categorisation extends from classification schemes used in previous studies^32,33^.

**Table 1:** Categories of recovery activity.

Category of recovery activities	Criteria—Message conveys information relating to one or more of the following:
Mental health and emotional support	Experiencing anxiety, PTSD, or mental health problems from the fires Providing Emotional support to bushfire victims Assistance with mental health
Post-disaster reconstruction and infrastructure services	Assessing or understanding the level of damage to physical infrastructure (e.g., homes, roads, electricity, gas, water). Note: This does not include tweets that talk generally about homes being burnt down without providing any specific information (e.g., address/area, image, etc.) to accurately identify the building(s) in question. Homes or infrastructure should be identifiable from the tweet or, at least, the tweet should lead people to understand the specific areas where homes have been damaged and reconstruction is needed. Otherwise, it does not support recovery through damage assessment or reconstruction activities Reconstruction of buildings or restoration of infrastructure services
Business & economic activities	Tourism, farming, or other business activities that support the economy of bushfire-affected communities
Environment	The condition of the environment such as bushland, street, air quality Clean up of the environment Planting of trees
Donations and financial support	Donations or financial support for bushfire-affected communities
Insurance claims	Insurance claims associated with bushfire damage
Animal welfare	The condition or welfare of animals
Solidarity and social cohesion	Fostering or hindering meaningful relationships between members of the community Behaving appropriately or inappropriately in times of disaster Maintaining or failing to maintain acceptable social standards in times of disaster Mutual support during disaster Antagonism or disagreement in times of disaster Social inclusion and a sense of belonging in times of disaster
Information support	How to gain an improved understanding of the bushfire situation How to make informed decisions about the bushfire situation How to be more prepared for future bushfires

**Table 2:** Categories of social media user (user groups).

Category of user	Description
Emergency agency	Government agencies responsible for helping communities to prepare for, prevent, mitigate the effects of, respond to, or recover from disasters
NGO/humanitarian	Non-profit organisations that aid vulnerable people and provide humanitarian assistance in times of armed conflict, famines, and natural disasters
News media	Encompasses journalists, reporters, news agents, and other media organisations involved in disseminating news and information to the public
Politician & political organisation	Includes politicians, elected public officials, political parties, special interest advocacy groups, and other formally organised associations aimed at achieving political agendas or influencing policy decisions
Business	Entities involved in trading or other commercial activities, including small private businesses and large corporations
Scientist & expert	Individuals with extensive training, expert knowledge, and insights to support decision making relating to the bushfires. Includes professors and distinguished academics, economists, medical experts, clinical psychologists, agricultural scientists, environmental consultants, structural engineers, meteorologists, etc
Celebrity	Famous individuals, especially in the entertainment industry, who attract public attention and have large numbers of social media followers
Community organisation	Community-based organisations established to provide services that build capacity, strengthen social connections, and improve the overall functioning of communities
Other government agency	Other government-owned organisations that provide public services that are not related to emergency management
Citizen	Ordinary members of the public who do not fall into any of the above categories

The entire sample of tweets were manually annotated. The possible categories to assign tweets were clearly defined (see Tables 1 and 2) so that all researchers can have a common understanding of how to annotate tweets. In categorising user groups and topics, each stage of the categorisation process was independently scrutinised by at least two researchers, ensuring that individual subjective bias was curtailed. Each researcher first assessed the tweets assigned to them for categorisation. To maximise accuracy of this task, the results were further scrutinised independently by at least two other researchers in the team. Where discrepancies existed, these were noted and later discussed amongst the entire research team to reach a consensus on the most appropriate categorisation for the tweets. The researchers acknowledge that a tweet could potentially have content that may be relevant to several aspects of disaster recovery. However, the rationale for the categorisation is that tweets should be assigned to just one specific category of disaster recovery, based on the core focus of the message or the aspect to which the content is most clearly relevant. This is helpful to understand which aspects of recovery have gained higher attention than others.

The researchers appreciate that there are existing topic modelling techniques and machine learning approaches for computationally extracting topics and classifying users^34–36^. However, the researchers have gone through the pain of manually annotating the data because of our research interest to further develop knowledge about the identified topics and user categories. Eventually, we hope to use the annotated data to train machining learning algorithms for automated classification of future data sets. This is ongoing method development research that is beyond the scope of the present study. In relation to the present study, the resultant data from the user and topic categorisation was subjected to a chi-square test of independence using SPSS software, to determine whether any statistically significant differences existed in how Twitter was used by different user groups to support different categories of disaster recovery over time^37^. The results were further analysed using measures explained in Table 3.

**Table 3:** Key measures that are vital for interpreting the results.

Measure	Explanatory note
%_wtn_mth (Percentage within month)	In relation to recovery activity, %_wtn_mth is the percentage of the total recovery-related tweets for any given month that relates to a specific recovery activity. This measure is important because it tells the extent to which each category of recovery activity is represented in the total recovery-related tweets generated for any given month Whereas, in relation to user group, %_wtn_mth is the percentage of the total recovery-related tweets for any given month that is generated by a specific user group. Similarly, this measure tells the extent to which each user group contributed to the total recovery-related tweets generated for any given month
%_wtn_group (Percentage within user group)	%_wtn_group is the percentage of tweets from a specific user group that is generated in a given month. This measure is vital because it tells how the total tweets from a specific user group is distributed across all the months investigated
%_wtn_rec (Percentage within recovery category)	%_wtn_rec is the percentage of tweets for a specific recovery activity that is generated in a given month. This measure is vital because it tells how the total tweets from a specific recovery activity is distributed across all the months investigated *Note* %_wtn_mth, %_wtn_group, and %_wtn_rec are relatable to the statistical concepts of 'percentage within column’ and ‘percentage within row’ that are commonly used in contingency tables^37^
Standardised residual	$Standardised\, residual=\frac{Observed-Expected}{\sqrt{Expected}}$ (See^37^ and^38^) This measure shows the strength of the difference between the observed and expected values We have used contingency tables to record and analyse the joint distribution of any two variables. For each contingency table, the count in a particular cell, ${x}_{ij}$ is the value of a random variable from *N* samples with a multinomial distribution^39^. Suppose that ${X}_{i}^{^{\prime}}$ represent the sum of counts in all cells along the *i*^th^ row, and ${X}_{j}^{^{\prime}}$ represents the sum of the elements in all cells along the *j*^th^ column. If the two variables involved in the Chi square test are independent based on the null hypothesis, then the expected value of the random variable ${x}_{ij}$ can be calculated using the formula^39^ $E\left({X}_{ij}\right)= \frac{{X}_{i}^{^{\prime}} {X}_{j}^{^{\prime}}}{N}$

## Results

### Temporal variations in bushfire recovery activity addressed on Twitter

**Figure 2:**
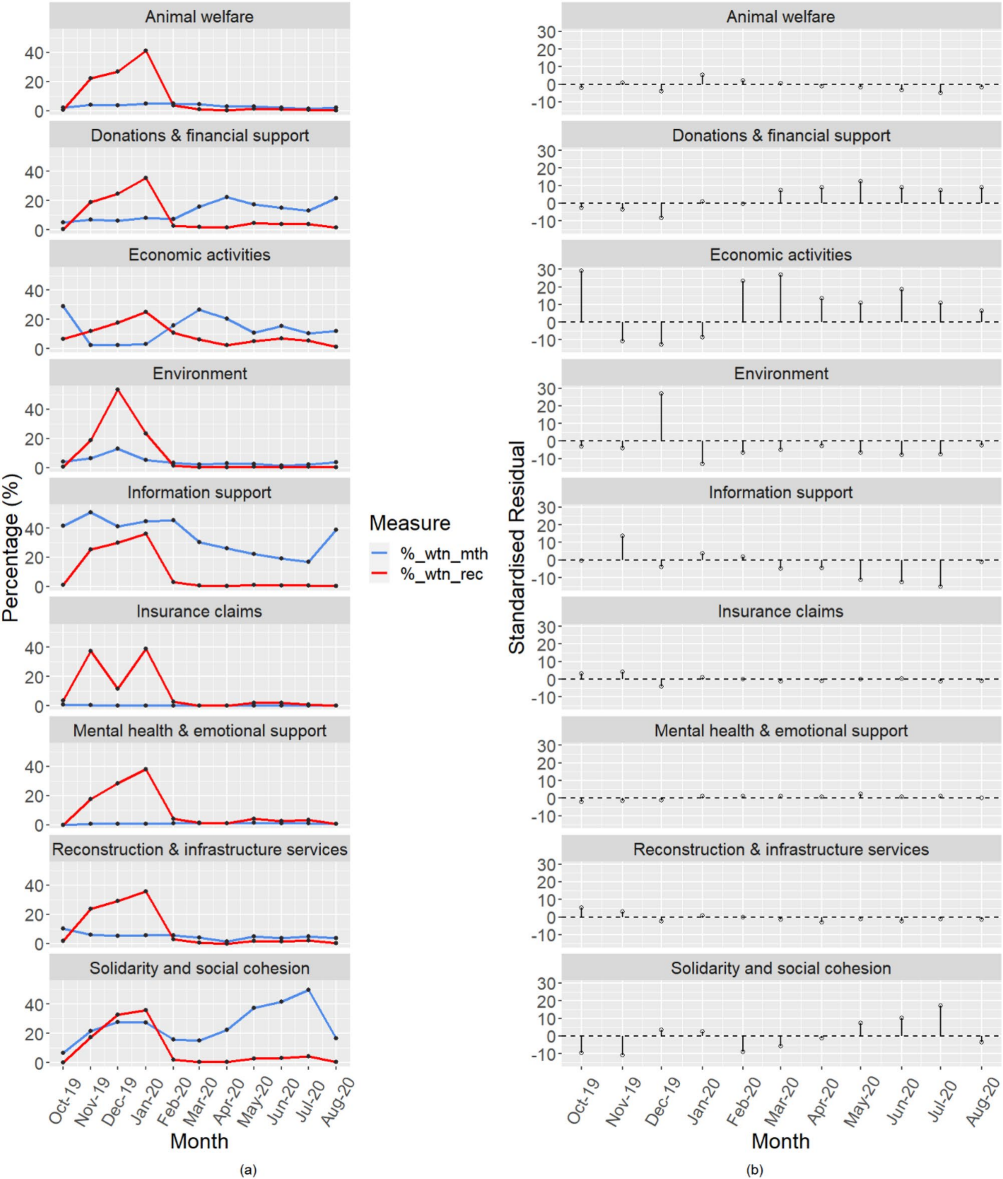
Monthly Twitter data for key categories of disaster recovery activities.

**Figure 3:**
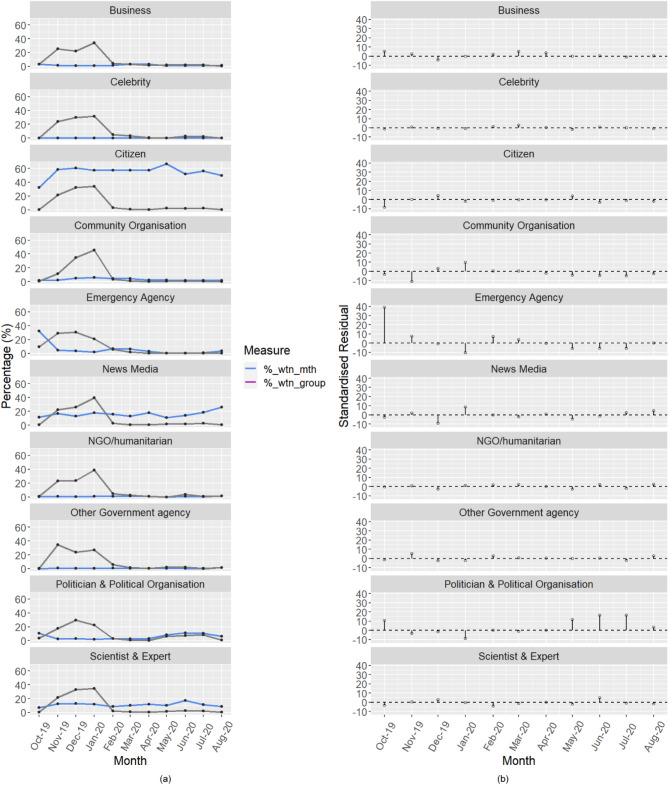
Monthly social media (Twitter) activities by various user groups.

A chi-square test of independence using the likelihood ratio revealed that, among the Twitter data included, tweets from different categories of recovery showed significant temporal variation, *X*^2^ (80, *N* = 61,362) = 5,508.69, *p* < 0.001. This is a moderate relationship as indicated by a Cramer's V value of 0.12^37,40^. As this is a multiple-comparison test, Bonferroni correction was applied, resulting in an adjusted alpha value of 0.0005^37^. Using this new adjusted alpha value, we established those relationships that were significant within the analyses. Figures 2 and 3 below summarise the variation in tweet volumes graphically. For example, Fig. 2a indicates that tweets in some topic categories were much more frequent than others. It also shows how different topic categories tended to peak at particular (and sometimes different) points of the recovery period studied. For example, information support messages accounted for approximately 41–51% of tweets on any topic for the months of October through February, peaking in November (see blue line for ‘information support’). By comparison, tweets about solidarity and social cohesion peaked much later as a proportion of all topics, accounting for 50% of all tweets in July 2020. To contextualise this variation, it should be noted that the monthly tweet rate varied greatly. Volumes in November, December and January together accounted for 87% of all 61,362 tweets in the study. Information support and solidarity & social cohesion were the most frequently discussed topics with 26,367 tweets and 16,288 tweets respectively, while mental health (388 tweets) and insurance claims (136 tweets) were the least discussed. Figure 2b displays the standardized residuals for each tweet topic category, where residuals below − 3 indicate that the observed count of tweets is significantly less than the expected value while residuals above 3 indicate that the observed count of tweets is significantly greater than the expected value. In the rest of this section, we report the statistically significant differences in tweet patterns by recovery activity, and briefly discuss some aspects of their interpretation.

The first set of results concerns tweets discussing donations and financial support. As Fig. 2a shows, December 2019 had one of the highest monthly tweets about donations and financial support (n = 1184). However, this number was found to be significantly lower (*p* < 0.0001) than the expected value of 1508. While not as obvious as December 2019, the donations and financial support tweets (n = 916) for the earlier month of November was also significantly lower (*p* < 0.0001) than the expected value of 1,026. Conversely, the proportion of donation and financial support tweets in the total recovery-related tweets for any given month was significantly higher between March and August 2020 (*p* < 0.0001), with May particularly recording a lot more tweets than expected.

It should be noted that the increase in donation and financial support tweets came in the lead-up to the 2020 Eden-Monaro by-election, held on 4 July 2020 to elect a Member of Parliament in the House of Representatives. Examining tweet content further showed that many of the tweets (see examples below) drew links between the by-election and the Government’s sudden announcement of financial support for bushfire-impacted communities in the Eden-Monaro electoral division. This highlights an important concern amongst members of the public that the timing of disaster recovery support was influenced by politicians’ interest in winning the favour of voters immediately before elections. This is consistent with previous studies, which have drawn strong links between the timing of public expenditure and elections in Australia and other countries^41–43^. After the elections, discussions about donation and financial support dropped markedly in August, but were still significantly more frequent than expected for that month. It should be noted, however, that most of these later tweets on donations and financial support had low engagement (i.e., likes, replies) and focused mostly on the unexpected donations from communities in Papua New Guinea as well as messages encouraging people to donate by purchasing the ‘Why Leave Town’ (WLT) Gift Cards, which can be issued as fathers’ day gift in support of bushfire victims and their communities. The WLT gift card is designed to support local communities as it can only be redeemed within the town it was bought.

Here we go.!! Suddenly bushfire effected "voters' in Eden Monaro are getting help!! I'm sure Carbargo "voters' will see thru this sudden 'rush' of help .. But… but…Where's the $2b in aid & all the donations of $m's gone? #ColourMeCynical

Who thinks Cobargo would have been getting all this attention from the federal govt, if there was no by-election coming up in Eden Monaco? (National Bushfire Recovery Fund spending plan is revealed – TODAY – after 5 mths inaction!) https://t.co/HHDGPhebxY

Oh.. How convenient that the Govt. waits till NOW to announce new bushfire funding (as opposed to, you know… when it was needed most!!!!) Obviously it's related to the upcoming #edenmonaro by-election. Sickening the Fed. Govt. waits till now to announce this! #theprojecttv

In the early phase of the bushfires (November 2019, December 2019, and January 2020), economic activity tweets were significantly lower (*p* < 0.0001) than expected. However, the proportion of economic activity tweets was found to be significantly higher in October 2019 as compared to other months (*p* < 0.0001). While economic activities represented, on average, 13.6% of the total recovery-related tweets for any given month, in October 2019 economy-related tweets accounted for 29.1% of all tweets. It is relevant to note that October 2019 was when the #buyfromthebush social media campaign was initiated to solicit support for rural communities by encouraging tourists and others to patronise businesses in communities impacted by record drought. In October 2019, the campaign went viral among Australian social media users and was used in several bushfire-related messages soliciting economic support for fire affected rural communities still recovering from the drought (see example tweets below). The #buyfromthebush social movement was quite influential in raising awareness for economic recovery of bushfire-impacted communities and led to the founder, Grace Brennan, winning the NSW Regional Woman of the Year award. Clearly, support for disaster recovery can be influenced by social movements of high interest to citizens.

After February 2020 when most of the fires had been extinguished, Twitter conversation about the bushfires dropped significantly. This drop in bushfire-related conversation came at the same time NSW was impacted by its first COVID-19 wave and lockdown, diverting attention away from the bushfires to COVID-related issues. However, the economic activity tweets between February and August 2020 were still found to be significantly higher (*p* < 0.0001) than expected. Notably, March 2020 recorded a disproportionately higher proportion of economy-related tweets, accounting for 26.9% of all tweets for that month. In March 2020, economic activities gained significantly higher attention amongst Twitter users, as they called for increased assistance to support bushfire-affected businesses now facing additional recovery challenges due to the COVID-19 lockdown (see example tweet below).

Have you seen the proliferation of the #buyfromthebush hashtag and associated accounts during the last two weeks? The Facebook and Instagram pages, founded by Grace Brennan, have gone from zero followers to 15,000 in just one week (now over 64 k and growing!) #ausag #agchatoz

Great Aussie spirit right here! Let’s help our struggling farmers and #buyfromthebush!

Bushfire communities were doing it tough before coronavirus. Now they’re being hit all over again. But the recovery has been too slow and assistance hasn’t gotten where it’s needed most. Today we met with businesses in Bega. We can’t forget them – and they can’t wait any more. https://t.co/dyBO6VI8ED

The proportion of environment-related tweets among all recovery-related tweets was significantly higher in December 2019 than other months (*p* < 0.0001), accounting for more than half (53%) of all tweets about the environment. Based on tweet content, this was mainly due to increased concern about secondary effects of the fires, including clean-up, smoke, visibility, poor air quality, and the impact on breathing (see examples of tweets below). All the other months, except October 2019, April 2020, and August 2020, recorded significantly lower environment-related tweets (*p* < 0.0001) than expected.

LOOK: Sydney, Australia has been shrouded in smoke for weeks from the New South Wales fires #NSWFires #AustraliaFires https://t.co/ms1cKMiijH

I find it so strange that everyone is casually going about their day as usual… When we're literally in a giant smoke haze, ash keeps falling from the sky, you can't see more than a block forward & every now & then you'll just cough up a giant ball of ashy mucus #NSWFires https://t.co/8lt5sG7l7T

Early months of the bushfires such as November 2019 and January 2020 had significantly higher (*p* < 0.0001) counts of information support tweets than expected whereas the later months from March to July 2020 recorded significantly lower (*p* < 0.0001) counts of information support tweets. Whilst information support remains the most frequent type of disaster recovery support on Twitter (see Fig. 2a), these results suggest that information support is more readily available on Twitter during the early phase of disaster recovery but diminishes with time. The topic of insurance claims recorded tweet counts that are not so different to the expectations across all months, except that the numbers of tweets were significantly higher (*p* < 0.0001) than expected in November 2019 and significantly lower (*p* < 0.0001) in December 2019. Animal welfare also recorded significantly lower (*p* < 0.0001) count of tweets than expected for December 2019 and July 2020. But the expected count of animal welfare tweets (n = 887) in January 2020 was significantly surpassed (*p* < 0.0001) with an observed value of 1049.

The proportion of reconstruction & infrastructure services tweets among all recovery-related tweets was significantly higher (*p* < 0.0001) in October 2019 compared to other months. On average, reconstruction & infrastructure services represented 5.2% of the total recovery-related tweets for any given month. However, the month of October 2019 recorded 10.5%. This was mainly due to heightened Twitter communication about the findings of an early building impact assessment conducted by government officials. In addition to damage assessment, there were also tweets focusing on the disruption and/or restoration of infrastructure services such as water, electricity, road network, housing, telecommunication, and internet. Several tweets conveyed information about the ‘Fire Up Cobargo Rebuild Festival’ to support reconstruction of damaged property in the town of Carbago, NSW.

Building Impact Assessment teams are working through fire affected areas, assessing the damage to properties. Assessments on the South Coast since 1 Jan confirm 449 homes destroyed, & more than 1,000 buildings saved. This work will continue over coming days. #nswrfs #nswfires https://t.co/zFeshcRPVo

There’s dirty water coming out of taps in Narrawallee because of the fires and power loss. Reminder for those to boil water and do whatever else you need to have clean water. #ClimateEmergency #ClimateChangeIsReal #NSWfires #AustraliaBurns https://t.co/nwP68oMqF4

Mike&Annie Cannon-Brookes pledge $12 m to install solar and battery systems in communities disconnected from the electricity grid by bushfire or flood. Resilient Energy Collective has already installed in Cobargo and Goongerah, thanks to 5B & Tesla

Solidarity and social cohesion tweets were observed to be significantly lower (*p* < 0.0001) than expected during the active bushfire period (October/November 2019) and towards the end of the period with active fires (February/March 2020). By contrast, at the peak of the bushfires (December 2019 and January 2020), solidarity and social cohesion tweets were significantly higher than expected, with *p* < 0.0001 and *p* < 0.0004 respectively. A provocative observation is that the proportion of solidarity and social cohesion tweets in the total recovery-related tweets for any given month was significantly higher (*p* < 0.0001) in May, June, and July 2020, after the active fires and response period. Content analysis showed that solidarity and social cohesion tweets sent during these months were dominated by reference to the Bushfires Royal Commission hearings, which included contentious discussion of bushfire cause and responsibility alongside calls for better action on climate change. Many of the solidarity and social cohesion tweets also raised concerns about failed governance and law to support climate change actions, including apportioning of blame or finger pointing. These discussions were sometimes contentious and socially divisive because of finger pointing and the politisation of climate change discussions. Twitter was used to hold governments accountable for perceived failures to act early in relation to climate change and to show responsiveness and leadership in times of crisis. Some example tweets are shown below.

@abcnews And next year she'll be changing state law to remove climate change as a consideration for new coal mine approvals. Enough is enough, the country will be uninhabitable if we're not careful! #ClimateEmergency #sydneysmoke #NSWfires.

#NSWfires Most local councils in NSW are so politically Green motivated that it is out of control and this years bush fire season has proved that local council law restrictions regarding the clearing of bush undergrowth on private and Crown land needs serious review.

### Temporal variations in user participation in bushfire recovery activities on Twitter

A chi-square test of independence using the likelihood ratio indicated that the disaster recovery-related messages posted on Twitter by different categories of users showed significant temporal variations, *X*^2^ (90, *N* = 61,362) = 6,362.01, *p* < 0.001. This is a moderate relationship as indicated by a Cramer's V value of 0.12^37,40^. A Bonferroni correction was applied, resulting in an adjusted alpha value of 0.0005^37^. Using this new adjusted alpha value, we established those relationships that are significant within the analyses. We outline these results by each user category.

Business users recorded significantly higher (*p* < 0.0001) number of tweets than expected during the early phase of the bushfires in October 2019 and in the early phase of COVID-19 wave in March and April 2020. Although January 2020 accounted for the highest count (34%) of all the recovery-related tweets posted by business users, this was consistent with expectation for the peak of the bushfires. Content analysis showed that business users were very active in disseminating messages of solidarity with bushfire-affected communities, including tweets with significant concern over the impacts of COVID-19 on the recovery process. Business users posted information about how they were assisting (e.g., helping to restore infrastructure services, providing financial support, assisting with insurance claims, discounting products and services for bushfire victims), as well as information on how others could assist. This result reveals that Twitter messaging for recovery is more likely to come from business users during the early phase of disasters, indicating that more effort may be required to sustain social media interest among business users as needed for supporting long-term recovery.

The proportion of tweets from citizens in any given month was significantly higher (*p* < 0.0001) in December 2019, accounting for 33% of all the tweets posted by citizens. Similarly, the proportion of tweets from community organisations in any given month was significantly higher (*p* < 0.0001) in December 2019 and January 2020, accounting for 35% and 46%, respectively, of all recovery-related tweets from community organisations. Both community organisations and citizens were actively providing information to support community members during December/January at the peak of the bushfires. Moreover, the proportion of tweets from citizens in the total tweets posted by users from all categories was significantly higher (*p* < 0.0001) in May 2020 compared to other months. On average, tweets from citizens represented 55% of the total tweets posted by users from all categories in any given month, whereas May 2020 recorded a significantly higher value of 67%. Tweet content indicated this was due to citizens’ responses to the Bushfires Royal Commission hearings, as well as increased concern that the timing of disaster recovery support was influenced by hidden agendas such as political interests amidst the 2020 Eden-Monaro by-election. However, the same could not be said for community organisations as their tweet counts for May (n = 26), June (n = 22), and July (n = 24) fell significantly below (*p* < 0.0001) the expected values of 58, 55, and 65 respectively.

The volumes of tweets posted by emergency agencies were significantly higher (*p* < 0.0001) than expected in the months of October 2019, November 2019, February 2020, and March. Tweets from emergency agencies were mainly emergency warnings and other risk management information for impacted communities. However, with the extinguishing of most fires and disaster recovery intensifying, emergency messages dropped significantly. For example, bushfire recovery-related tweets from emergency agencies in May 2020 (n = 5), June 2020 (n = 6), and July 2020 (n = 11) were significantly below (*p* < 0.0001) the expected values of 45, 43, and 50 respectively. Overall, emergency agencies were more active on Twitter during the early phase of the disaster recovery, but their contribution diminished significantly in the later period. Observed contributions from other government agencies were not so different from the expected values, except in November when expectation was significantly (*p* < 0.0001) surpassed.

Tweets from politicians and political organisations accounted for a significantly lower proportion of all tweets (*p* < 0.0001) in November 2019 (2.4%) and January 2020 (1.9%) compared to other months (see Fig. 3). The proportion of tweets from politicians and political organisations was also low (2.8%) in December 2019, but this did not reach statistical significance at *p* = 0.0916. However, the figures were higher in October 2019 (10.4%), May 2020 (8.6%), June 2020 (11%), and July 2020 (10.4%), and these differences reached statistical significance (*p* < 0.0001). Although politicians and political organisations recorded more tweet counts in December (n = 539) than in any other month, the results indicate that politicians and political organisations were in effect less engaged with the public than expected at the peak of the bushfires and more active on bushfire recovery-related conversations at other moments of interest such as the period of the 2020 Eden-Monaro by-election and the Bushfires Royal Commission hearings.

Celebrities as well as NGOs and humanitarian organisations posted recovery-related messages in a manner that is not so different from the expected volume of tweets across all months. Just before the COVID-19 lockdown, celebrities used Twitter to communicate messages of hope, including information about concerts and upcoming events to raise donations in support of bushfire-impacted communities (see example tweet below). Similarly, scientists and experts participated as expected, except that the tweet counts were significantly lower (*p* < 0.0001) than expected in October 2019 and February 2020 and significantly higher (*p* < 0.0001) in June 2020 (see Fig. 3). For news media, participation level was significantly lower (*p* < 0.0001) than expected in December 2019 during the peak of the bushfires. However, new media contributed actively to keep communities informed of the bushfires and matters relating to the recovery, particularly in January 2020, which contained 40% of all recovery-related tweets posted by news media.

Played a surprise show in Bega last night for the community that’s been through a very hard time. I still watch the bushfire vision and feel so sad for our country. I hope I gave you a night to release your mind and smile again.

### User group contribution levels on Twitter vary across bushfire recovery activities

A chi-square test of independence using the likelihood ratio showed that the volume of recovery-related messages posted on Twitter during and after the bushfires varied significantly by user category, *X*^2^ (72, *N* = 61,362) = 4,698.89, *p* < 0.001. This is a moderate relationship as indicated by a Cramer's V value of 0.12^37,40^. Bonferroni correction was applied, resulting in an adjusted alpha value of 0.0005^37^. Using this new adjusted alpha value, we established which relationships were significant within the analyses. We outline these results by user category.

**Figure 4:**
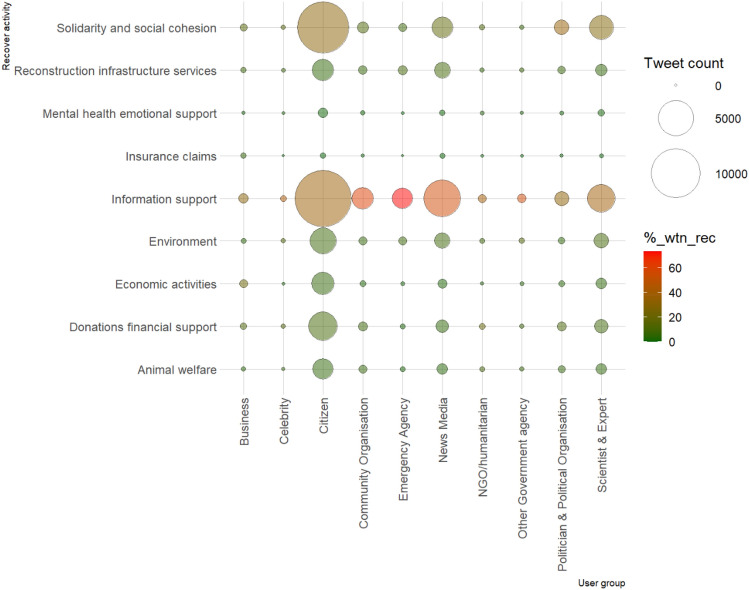
User group contribution to different aspects of disaster recovery.

**Figure 5:**
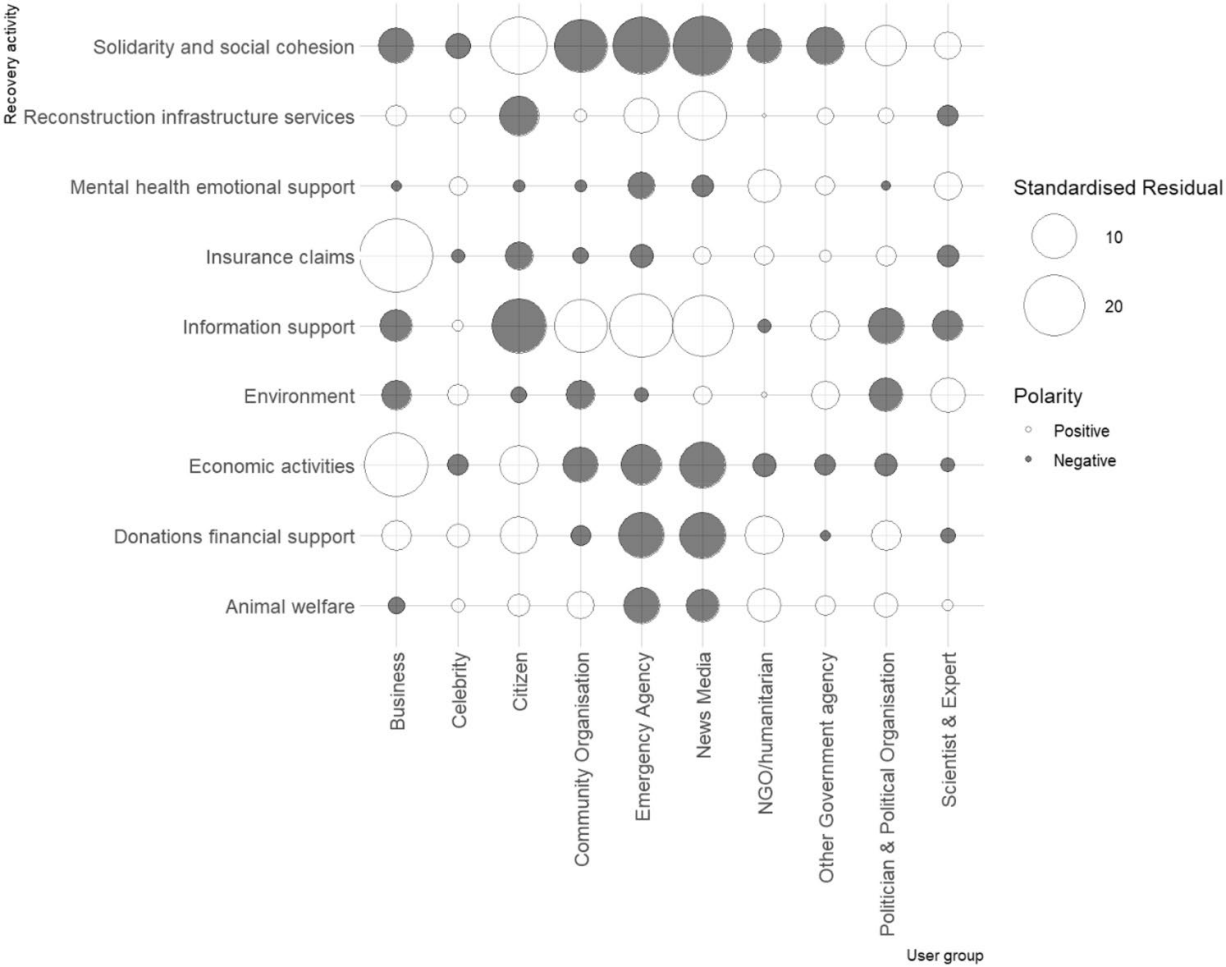
Standardised residual for user group participation levels across recovery activities.

Citizens were found to have contributed a significantly higher (*p* < 0.0001) proportion of tweets than expected in relation to solidarity and social cohesion, economic activities, and donations & financial support. While tweets from citizens represented 57.1% of all tweets in any given topic category, this group posted 69.4% of all tweets in the category of economic activities, 68.6% of all tweets about solidarity and social cohesion, and 65.9% of all tweets about donations & financial support (Fig. 4). However, as reflected in Fig. 5, citizens’ tweets on information support (n = 13,440), reconstruction & infrastructure services (n = 1,669), and insurance claims (n = 47) were significantly lower (*p* < 0.0001) than the expected values of 15,406, 2,018, and 80 respectively. Community organisations were more likely to contribute to information support than other recovery activities as 61.2% of all tweets posted by community organisations provided information support. Although, community organisations contributed significantly lower (*p* < 0.0001) than expected for economic activities, environment, and solidarity and social cohesion.

The proportion of tweets from emergency agencies was significantly higher (*p* < 0.0001) for information support than other categories of recovery activities. Information support accounted for 73.5% of all recovery-related tweets posted by emergency agencies. These posts were mainly emergency warnings and bushfire updates. The number of reconstruction & infrastructure services tweets (n = 187) were also significantly higher (*p* < 0.0001) than expected (n = 120) for emergency agencies. However, emergency agencies’ tweets about animal welfare (n = 27), donations & financial support (n = 29), economic activities (n = 14), mental health & emotional support (n = 1), and solidarity & social cohesion (n = 152) were significantly below (*p* < 0.0001) the expected values of 86, 166, 94, 13, and 564, respectively (see pattern in Fig. 5).

Like emergency agencies, the proportion of tweets from news media in any given category of recovery activities was significantly higher (*p* < 0.0001) for information support than other categories. Information support accounted for 56.2% of all the recovery-related tweets posted by news media. It was not uncommon for news media to retweet or post emergency information received from emergency agencies. News media also made a significantly higher contribution to reconstruction & infrastructure services (*p* < 0.0001), making this group exhibit very similar participation pattern with emergency agencies. Like emergency agencies, news media’ tweets about animal welfare (n = 304), donations & financial support (n = 460), economic activities (n = 209), and solidarity & social cohesion (n = 1,626) were significantly below (*p* < 0.0001) the expected values of 407, 765, 434, and 2,592 respectively (see pattern in Fig. 5).

NGOs and humanitarian organisations contributed only a small number of tweets (354) overall, yet some distinctive patterns in their practice were seen. NGOs and humanitarian organisations contributed significantly higher (*p* < 0.0001) than expected for animal welfare, donations & financial support, and mental health & emotional support. However, solidarity and social cohesion tweets were significantly lower (*p* < 0.0001) than expected from NGOs and humanitarian organisations. Turning to other government agencies, this group contributed significantly more messages (*p* < 0.0001) about the environment (n = 42) and information support (n = 182) than the expected values of 24 and 138 respectively. However, solidarity and social cohesion tweets (n = 20) were significantly lower (*p* < 0.0001) than expected (n = 86) from other government agencies.

Politicians & political organisations contributed a significantly higher (*p* < 0.0001) proportion of solidarity and social cohesion tweets as compared to other recovery categories, accounting for 36.4% of all tweets from this group. They also contributed strongly to donations & financial support with 192 tweets, significantly higher (*p* < 0.0001) than the expected value of 143. However, unlike other government agencies, politicians & political organisations posted significantly fewer tweets (*p* < 0.0001) about the environment (n = 75) and information support (n = 603) than the expected values of 138 and 783 respectively (see Fig. 5). Scientists & experts contributed significantly more tweets (*p* < 0.0001) than expected towards solidarity and social cohesion, environment, and mental health & emotional support, but significantly less so (*p* < 0.0001) towards information support. Another interesting observation is that businesses contributed significantly more tweets (*p* < 0.0001) than expected towards aspects of recovery that directly deal with finance, including insurance claims, economic activities, and donations & financial support. Whereas the number of tweets posted by businesses were significantly lower (*p* < 0.0001) than expected for information support, environment, and solidarity and social cohesion.

## Summary of key findings and discussion

- The timing and extent of participation on Twitter are not homogenous in terms of disaster recovery activities (i.e., tweet topics), but rather vary by user group.
- The timing of disaster recovery support on Twitter can be influenced by key events beyond the disaster, such as elections, public hearings, business initiatives/social movements (#buyfromthebush), and impact reports.
- Overall, Twitter activity around the bushfires dropped significantly over time, particularly after the active phase of the disaster. This finding is consistent with previous studies which report that social media use for disaster recovery is challenging to study because the tail off in the volume of data prevents meaningful analysis^8,16^.
- Emergency agencies were found to be more active on Twitter during the early phase of the disaster, contributing significantly towards information support as well as reconstruction and infrastructure services. They were particularly involved in emergency warnings and updates about active bushfires. However, their contribution diminished significantly in the later recovery phases as general warnings and communication with communities at risk died down. This observed pattern may well reflect the legal mandate and expectation of emergency agencies to provide emergency response and relief, not so much on recovery.
- Businesses made significant contributions to topics directly involving finance such as donations and financial support, assistance with insurance claims, and economic support, including messages about discounting products and services for bushfire victims.
- Overall, both citizens and news media recorded strong contributions to all categories of recovery activity compared to other groups. However, citizens were more likely to contribute messages about solidarity & social cohesion, economic activities, or donations & financial support. Community organisations were more likely to contribute to information support than to other recovery activities.
- Emergency agencies and news media exhibited very similar patterns of participation, including in relation to the aspects of recovery in which they recorded significantly higher levels of contribution and those in which they recorded significantly lower number of tweets than expected.
- Information support was the most available bushfire recovery support on Twitter, particularly in the early phases, followed by solidarity & social cohesion. By contrast, claiming on insurance was the least supported recovery activity on Twitter. It should be noted, though, that information support diminished significantly in the later phase of the recovery timeline.

In this paper, our account of ‘group’ variation on Twitter is organised around longstanding and recognisable demographic segmentations of society, and it has generated statistically significant results which appear informative for disaster management practice. Zhang et al.^44^ argue that such approaches “might not fully reveal the formation and distribution of public opinion” on social media and that we should favour dynamic “murmuration” patterns among users that resemble the changing flocking patterns of birds. The need to consider networks of actors becomes more appealing if we consider that in today’s digital spaces or online information environment, like-minded citizens and people from the same social groups are often densely connected and express highly consistent messages internally yet quite distinct from each other^44^. Similarly, researchers might consider how users actively perform “ambient affiliation” by aligning or disaligning around proposed interpersonal “bonds”^45^. We agree with the view that homogeneity and difference in behaviour need not stem only from pre-defined functional roles in society, and we are currently conducting ‘affiliation’ analyses of our Twitter data, which we see as complementary to the analyses presented here.

Our results on temporal variation in Twitter activity around the Black Summer bushfires suggest that here, too, a complementary approach is ideal. We can think in terms of discrete predictable phases but, we must also recognise the multidimensional nature of each phase^28^ and the potential for overlap or layering of multiple phases and multiple crises over each other^46^. Communities can even “reverse directions along the progression cycle”^47^ and a community experiencing the beginning of the COVID-19 pandemic on the back of bushfire devastations would be one of the most likely to experience such a reversal. So, although a fairly simple sequential model can show us patterns that seem to have practical as well as statistical significance, we still need to unpack a lot more about what it means for a disaster precinct to be in ‘recovery’.

An important step is to consider whether a region such as the NSW South Coast could be expected to move in and out of disaster phases as one community and, if not, how should future research account for this? One possibility would be to add the dimension of ‘place’ to analyses of temporal variation among groups and topics, in order to help distinguish places within the region that were genuinely in recovery from those still in response and so capture more clearly the “recovery” characteristics of the posts analyzed. Not including such a fine grained place analysis could be considered a limitation of the present study, however there are many challenges to undertaking such an analysis that researchers need to address.

The first problem for place analysis is data sufficiency—a place analysis would require location data at a level of aggregation lower than region. In our 61,362 tweets this did not occur often enough to provide a sample large enough for the required statistical tests, making it difficult to integrate place variation with topic and group variation analyses.

Moreover, the level of aggregation meaningful enough for capturing whether one place was in recovery while another was not in recovery is likely to be very ‘micro’, based on our experience as residents and/or on personal communication with residents. In the Black Summer fires, one property might have been devastated while their neighbour just across the creek was spared, and many families evacuated more than once or went on high alert multiple times, whether or not they eventually suffered fire damage^48^. Equally, it is possible that two neighbours had fire damage, but one neighbour might be in quite a different part of the recovery phase from another, depending on the type and extent of damage, their family or workforce structure, humans/pets/farmed animals harmed, type of buildings that need reconstruction, where such materials would need to come from and how long their response queue was, and so on.

To inform a place analysis, tweet content would therefore need to identify street intersections, property block numbers, unnamed hills, one side of a river, etc.: this was seldom found in our data. Alternatively, the geographical identification metadata could be used, if there are adequate geotagged tweets, but, as mentioned earlier, only 1% of tweets are geotagged.

Thinking about place in this way brings us back to our earlier questions about groups and communities. We must consider *for whom* some geospatial location counts as a ‘place’, since place involves connection and experience^49^. In analysing recovery or its coverage on Twitter, we will sometimes be interested in the individual, other times a family, a business, the street, the village; perhaps a community of cyclists, teachers or farmers; or some emergent community of people who ‘flock’ around a particular hashtag. Future work should consider how to operationalise place analyses in social media research, but just as there is no simple movement from hazard mitigation to preparedness, to response, then to recovery activities, there may be no single way to frame a place analysis that serves all purposes.

## Practical implications and concluding remarks

A novel finding from this study is that Twitter use for bushfire recovery is not homogenous but appears to reflect variation in habitus among different communities of practice that are constituted independently of their participation on Twitter platform. This is an important empirical contribution to a growing body of evidence that public opinion on social media platforms vary with user characteristics, further helping to establish the much-needed theoretical foundation to nurture the growth of this research area. Evidence from previous studies have identified political affiliation, age, race/ethnicity^50^, online social connections^44^, and affiliations to echo chambers^51,52^ as user characteristics that can influence public opinion on social media platforms. The present study has shown that, in the context of disaster recovery, the timing and topic of social media public discourse can also vary according to professional affiliation (e.g., scientists & experts, celebrities, news media, NGO/humanitarian organisations, emergency agencies, etc.). We believe that by building knowledge about how different clusters of social actors express bushfire recovery-related views and information on social media over time, we are establishing contours of knowledge that will be useful to emergency management practitioners and researchers alike, and helping to build this field. In particular, our approach of using recognisable user groupings, rather than emergent groupings, should provide immediately accessible information to support emergency management practitioners involved in disaster communication strategies at community or local government level, as well as state and national government level.

Another key finding from this research is that emergency agencies do not contribute much, on Twitter, to the later phase of recovery once the need for emergency response and relief has ended. This suggests the need to have other recovery-specific agencies, with noticeable social media presence, to bridge the gap and ensure that there is a continuum of online support as individuals and communities transition from the disaster response phase to the recovery phase. Our research findings also suggest a need for disaster recovery agencies to consider how key events such as elections, public hearings, and online social movements may sway social media public discourse on recovery topics and how that might affect the sentiment, morale, or recovery of individuals and communities that consume such online contents.

The study has also confirmed that the tail-off in social media activity seen in other crisis contexts also occurs in bushfires and in the Australian geographical context, suggesting a need to focus on whether there is something that researchers and practitioners can do to extend tweeting activity into the period relevant for fostering longer term disaster recovery. The findings imply a need to understand the distinct interests of relevant user groups, so as to better target effort in improving (or sustaining) their participation in social media recovery activities. As a research community, we have an opportunity to address this need by harnessing real-time data to understand changing community sentiment and other important dimensions of the recovery process. It is therefore our recommendation that future research seeks to 1. further investigate the peculiar interests and affective orientation of different user groups, 2. identify ways to better engage and sustain the participation of different user groups in social media activity around disaster recovery, and 3. extend this research through a comparative analysis to investigate whether there could be any temporal, topical, or group variations (or otherwise) for other social media and disaster contexts, such as Facebook, or floods, in other regions or nations.

To improve user participation in the recovery phase, we recommend that recovery agencies purposefully and actively develop solutions for utilising Twitter as a strategic tool for monitoring and fostering recovery from disasters. Twitter data should be mined for real-time monitoring to gain insight into the aspects of disaster recovery that are salient, neglected or otherwise requiring attention. This will benefit from future research to automate, through machine learning algorithms, the classification of users and disaster recovery content. Strategies should aim to employ social media for online promotion of recovery activities, ensuring strong engagement with a diverse range of user groups. For example, staff should be trained in how best to drive community participation and promote recovery-related content that can effectively engage different user groups, using the findings from this study as a guide to the key aspects of disaster recovery of interest to different user groups. User engagement can be fostered by capturing and sharing inspiring stories and experiences of survivors on social media to motivate actions in support of recovery activities. Strategies should include the use of viral videos, testimonials/recovery progress, and other media content that can draw the focus of the global community of supporters and volunteers to the needs of affected communities.

## Data Availability

The sample dataset analysed during the current study are not publicly available due to the risk of inadvertent disclosure of identifiers and ethical issues arising from associating research findings to specific individuals or entities. However, the data may be available from the corresponding author on reasonable request.
